# Hysteresis in Organic Electrochemical Transistors: Distinction of Capacitive and Inductive Effects

**DOI:** 10.1021/acs.jpclett.3c03062

**Published:** 2023-12-01

**Authors:** Juan Bisquert

**Affiliations:** Institute of Advanced Materials (INAM), Universitat Jaume I, 12006 Castelló, Spain

## Abstract

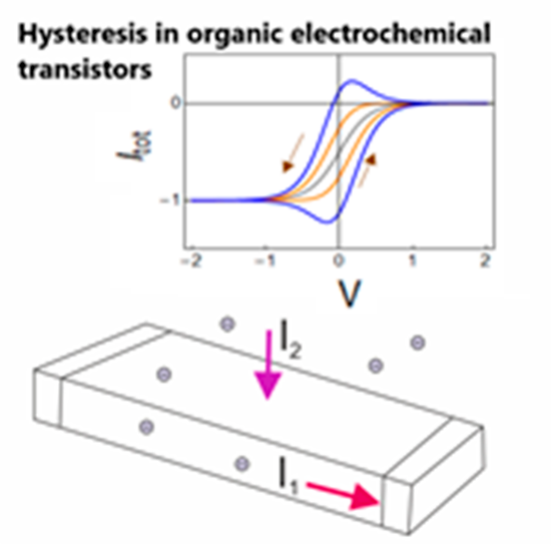

Organic electrochemical transistors (OECTs) are effective devices for neuromorphic applications, bioelectronics, and sensors. Numerous reports in the literature show persistent dynamical hysteresis effects in the current–voltage curves, attributed to the slow ionic charging of the channel under the applied gate voltage. Here we present a model that considers the dominant electrical and electrochemical operation aspects of the device based on a thermodynamic function of ion insertion. We identify the volume capacitance as the derivative of the thermodynamic function, associated with the chemical capacitance of the ionic–electronic film. The dynamical analysis shows that the system contains both capacitive and inductive hysteresis effects. The inductor response, which can be observed in impedance spectroscopy, is associated with ionic diffusion from the surface to fill the channel up to the equilibrium value. The model reveals the multiple dynamical features associated with specific kinetic relaxations that control the transient and impedance response of the OCET.

Organic electrochemical transistors (OECTs) have gained considerable attention due to their unique capability to transduce both electronic and ionic signals,^1,2^ making them well-suited for various applications in the realms of neuromorphic systems,^3−6^ bioelectronics,^7,8^ and sensors.^9^ These versatile three-terminal devices consist of an organic mixed ionic electronic conductor (OMIEC)^10^ serving as the channel connecting the source and drain electrodes. An electrolyte functions as the ion reservoir positioned between the channel and the gate electrode.^11^

In OECTs, the control of the drain–source bias (*u*_*d*_) remains constant, while variations in the gate–source voltage (*V*) govern the flow of mobile ions between the OMIEC channel and the electrolyte. This modulation of the ions leads to changes in the doping states and the conductivity of the OMIEC channel. Consequently, OECTs are volumetric devices, endowing them with high transconductance, which results in substantial amplification capabilities and enables them to operate effectively at relatively low voltages.

To measure the current–voltage characteristics a voltage scan of the form
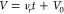
1is applied. The parameter *v*_*r*_ is the voltage sweep velocity, and *V*_0_ is the initial voltage. One considers a negative (reverse, *v*_*r*_ < 0) sweep followed by a positive sweep (forward, *v*_*r*_ > 0) that returns to the starting voltage. Characteristic results by Leo and co-workers are shown in Figure 1.^12^ An important hysteresis effect is observed, i.e., a mismatch between the forward and backward scans in the transfer curves. Hysteresis has been reported widely in OECTs^11,13,14^ and attributed to a delay of ion charging.^15^

**Figure 1:**
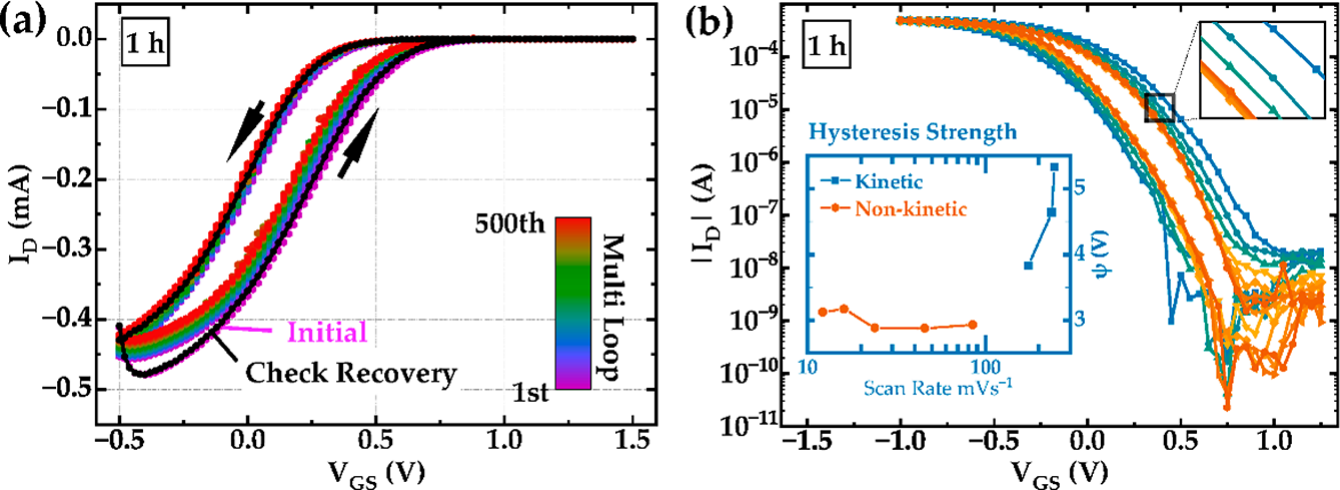
(a) Bias stress stability measurement in an OECT where the PEDOT:Tos film is annealed for 1 h at 70 °C. The measurement spans approximately 6 h, with a recovery measurement showing no evidence of device degradation. (b) Demonstration of a scan-rate-dependent transfer curve measurement where the inlet shows the hysteresis strength ψ. The channel width (*w*), length (*L*), and gate distance are 150, 150, and 50 μm, respectively. The film thickness (*d*) for the bias stress measurement is 80 nm and for the scan-rate-dependent measurement is 119 nm. The drain–source voltage (VDS) is kept constant at −0.2 V. The scan speed for bias stress measurement is ∼180 mV s^–1^. Reproduced with permission from Shameem, R.; Bongartz, L. M.; Weissbach, A.; Kleemann, H.; Leo, K. *Applied Sciences***2023**, 13, 5754; licensed under a Creative Commons Attribution (CC BY 4.0) license.^12^

In this work, we present a model that accounts quantitatively for different hysteresis effects in OECTs. We start from the basic framework for the transistor current established by Bernards and Malliaras,^16^ and we consider some additional key aspects. First, disorder produces a broad density of states (DOS) in the OMIEC, which can be treated experimentally from the properties of the chemical capacitance.^17,18^ In addition, we use previous insights on the characterization of inductive and capacitive hysteresis that have been described for perovskite solar cells and memristors.^19−22^ This model allows us to classify different types of hysteresis under forward and back sweep cycles. In addition, we develop the associated model of impedance spectroscopy that produces a complete diagnosis of the inductive features due to transport effects. We assume *u*_*d*_ is small, enabling essentially uniform hole carrier density *p* in the channel. A number of advanced modeling methods can be applied to simulate realistic transistor measurements (inhomogeneous carrier distributions, depletion, saturation current, lateral currents, traps, swelling, and so forth),^23−26^ but here we reduce the geometrical features to the simplest situation in order to obtain the main physical causes of the transient dynamics inherent to the OECT configuration.

We remark that Figure 1 shows two distinct types of hysteresis. Kinetic hysteresis depends on the scan rate, and the extent of hysteresis is enhanced for faster scans. This behavior is the topic of this Letter. On the other hand at slow scan rates there is a bistable behavior that will not be considered here.

According to the scheme in Figure 2 there are two components of the current.^16^ The first is the hole current across the channel
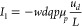
2Here *q* is the elementary charge and μ_*p*_ is the mobility. The density of holes is produced by the insertion of anions with volume density *X*; hence
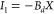
3where we have defined
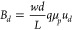
4

**Figure 2:**
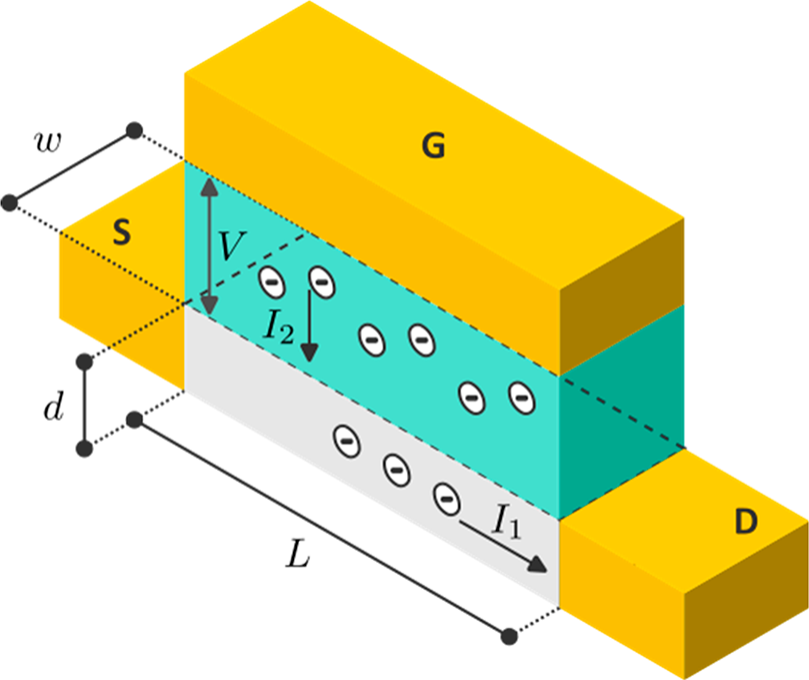
Scheme of the operation of the OECT. The green zone is the electrolyte, the gray zone is the OMIEC layer, and the yellow zones are the gate (G), source (S), and drain (D) electrodes. The anions inserted into the film are the source of electronic holes that carry the current.

The second component of the current is caused by the insertion of cations from the electrolyte by the gate voltage that produces the entrance of the same number of holes from the S electrode
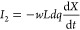
5

In total we have the conduction current
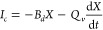
6where *Q*_*v*_ = *wLdq*. Equation 6 is equivalent to that formulated by Bernard and Malliaras.^16^

Another important effect is the role of the electrolyte. The applied voltage *V* is divided into two components
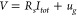
7*R*_*s*_ is the series resistance of the solution, and *u*_*g*_ is the voltage applied to the organic film, which causes a change of the Fermi level (electrochemical potential). The total current is
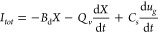
8Here we have added to *I*_*c*_ the capacitive current due to the charging of the capacitance *C*_*S*_ at the solution/film interface, which has a constant capacitance similar to a Helmholtz capacitance.

As mentioned, a change in *u*_*g*_ modifies the amount of inserted anions. In equilibrium, there is the thermodynamic function *X*_*eq*_(*u*_*g*_) that states the extent of intercalation of ions according to the voltage in the film. This function can be obtained by different electrochemical methods,^27^ and it has been amply studied for conducting polymers (with the statistics of polarons and bipolarons)^28,29^ and organic transistors.^30,31^ As a significant example of the ionic thermodynamic function we recall the Frumkin function that has been widely used in Li intercalation studies:^32,33^
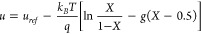
9Here *k*_*B*_*T* is the thermal energy and *g* is the adimensional interaction parameter that accounts for the interactions between intercalated ions in the mean field approximation.^34^

Another quantity of great significance for the charging properties is the chemical capacitance of the ions.^17,18^ This is obtained by the derivative of the thermodynamic function, with respect to the electrochemical potential.
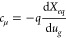
10For example, the Frumkin model of eq 9 gives the following result
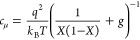
11The quantity *c*_μ_ is a volumetric density, and the total chemical capacitance is
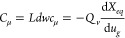
12

The chemical capacitance is readily measured by voltammetry or impedance spectroscopy.^35−37^ Indeed, the extent of charging of electrochemical transistors is usually obtained by an integration of the chemical capacitance, as follows
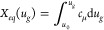
13On the other hand, the charging of the polymer film can be stated in terms of the DOS *g*(*E*) as a function of energy *E*
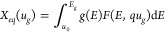
14where *F*(*E*, *qu*_*g*_) is the Fermi–Dirac thermal occupation function for electrochemical potential *E*_*F*_ = *qu*_*g*_. Applying eq 10, it is obtained^36,37^
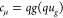
15Therefore, the chemical capacitance provides a direct measure of the DOS. Usually in organic conductors the measured capacitance shows very broad features.^35,38^ The dispersion of the DOS is attributable to disorder in the form of Gaussian functions and additional features.^17,31,37,39^ It has been well-recognized that the volume capacitance C* used in OECT is a strong function of the gate voltage, often displaying peak values.^2^ This is an intrinsic property of the chemical capacitance simply due to the thermodynamic function, e.g., see eq 11.^33^ It is only natural to identify the volumetric capacitance C* to the more general concept of chemical capacitance *C*_μ_.

The next aspect of the transient current is the transport of ions from the insertion at the solid/electrolyte interface until the organic layer is homogeneously filled according to the potential *u*_*g*_ in the film. Here we assume that the determinant process is the diffusion of ions with a chemical diffusion coefficient *D*_*X*_.^40^ According to Fick’s law and the conservation equation, for a species of concentration *n* we have
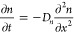
16

Instead of solving the full transport model, we use the approximation to eq 16 that contains the main dynamical properties:
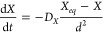
17The charging process will be complete when *X* = *X*_*eq*_ is everywhere in the organic layer. We express eq 17 as
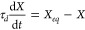
18where
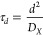
19is the characteristic time for vertical ionic diffusion along the film thickness.

During the diffusive charging, the concentration is inhomogeneous in the vertical direction. Bernard and Malliaras^16^ introduce a factor *f* ≈ 1/2 that gives the average of the mobile charge along the film thickness. However, since the problem can be solved rigorously by using eq 16, we do not introduce such a factor.

Now eqs 7, 8, and 18 form a complete system that can describe the transient behavior of the organic transistor in the the measurement conditions that have been commented in the introduction. We remark that eq 18 has the structure of a chemical inductor.^41^ When combined, eq 8 and 18 form a system that has been described for the hysteresis properties of halide perovskite devices.^42^

In order to illustrate the dynamical properties of the model we choose the following form for the equilibrium function:
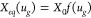
20Here, *X*_0_ is a maximal density and
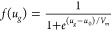
21is a function that varies between 0 and 1. *u*_0_ is the voltage of half occupancy, and *V*_*m*_ is a voltage that indicates broadening of the distribution. This function is chosen for convenience, to show the dynamical properties of the model. For the realistic thermodynamic functions we refer the reader to the literature mentioned above.

Having set the form of the thermodynamic function, we can illustrate the different properties of the model. First, we note that the equilibrium current is
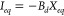
22This is shown in Figure 3a. In the simulations, we use numbers without units since the point of the study is to establish the main classes of hysteresis. The calculation of the chemical capacitance provides the result
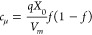
23This is shown in Figure 3b. The shape is a peak that approximates the characteristic behavior of capacitance in OCETs.^2^

**Figure 3:**
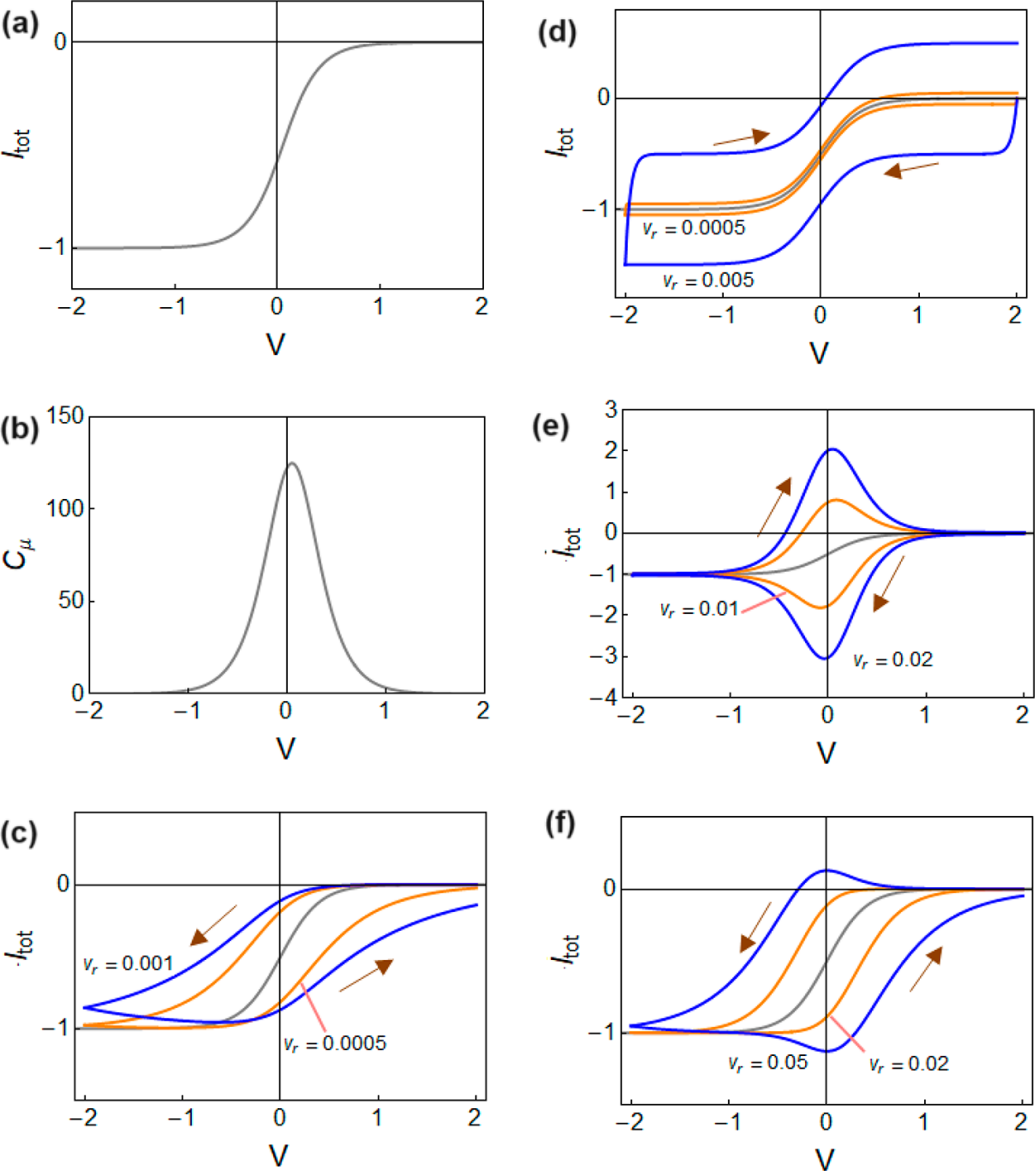
(a) Current–voltage curve and (b) chemical capacitance. Parameters: *L* = 10, *d* = 0.1, *w* = 1, *X*_0_ = 100, *u*_0_ = 0, *V*_*m*_ = 0.2, *qμ*_*p*_*u*_*d*_ = 1. Panels c–f show current–voltage curves at different voltage sweep rates (*v*_*r*_) and the reference equilibrium curve as in panel a. (c) Hysteresis due to series resistance, *R*_*s*_ = 1, *C*_*s*_ = 1000, *X* = *X*_*eq*_. (d) Hysteresis due to constant capacitance, *R*_*s*_ = 0.1, *C*_*s*_ = 100, *X* = *X*_*eq*_. (e) Hysteresis due to chemical capacitance, *R*_*s*_ = 0.1, *C*_*s*_ = 1, τ_*d*_ ≈ 0. (f) Hysteresis due to inductive effect of ions, *R*_*s*_ = 0.1, *C*_*s*_ = 1, τ_*d*_ = 100.

We remark that for any form of the *X*_*eq*_(*u*_*g*_), the number of carrier increases with the voltage, due to eq 13, and the equilibrium current increases with the voltage, as found also in most literature reports such as those of Figure 1.^12,14^ However, it has also been reported in the literature that the current decreases with the voltage,^38^ causing a negative resistance that is important for artificial neuron systems.^5,43^ This effect is often due to a mobility that depends on the voltage (or the carrier concentration)^17,18,29,44^ and will not be further considered here, as we restrict our attention to a system with constant hole mobility, μ_*p*_.

Based on the general equations of the model, we explore the different causes of hysteresis.

We first assume that ion charging is fast so that *X* ≈ *X*_*eq*_ and neglect the series resistance. Then we have
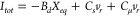
24This result shows two terms of purely capacitive hysteresis. The current is higher than equilibrium current for the forward sweep and lower for the reverse sweep. For the constant capacitance the current is shown in Figure 3d. On the other hand the capacitive current due to charging the chemical capacitance is shown in Figure 3e. We observe that the voltammetry records the DOS of Figure 3b. This result is amply reported in the literature as commented before.^31^ In Figure 3e the capacitive current is added to the drift current. To measure the chemical capacitance alone, we can simply set *u*_*d*_ = 0 to suppress the lateral current. It is also important to remark that capacitive current is proportional to the scan rate, as is well-known in electrochemistry.^45^

Next we ignore the capacitive effect but introduce a considerable series resistance. By eqs 7 and 8 we obtain
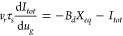
25Here we have defined the time
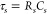
26Equation 25 indicates that the current requires a time τ_*s*_ to obtain stationary value *I*_*eq*_. The resulting hysteresis behavior is shown in Figure 3c. We remark that hysteresis is inverted with respect to capacitive hysteresis, with the forward current being smaller than the reverse current.

Finally we consider the effect of all the terms, including ion diffusion, so that *X* is a variable now. The current equation under constant sweep rate is
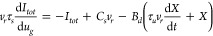
27Here we have introduced the time
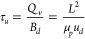
28Clearly τ_*u*_ is a transit time of the lateral drift transport of holes along the channel distance *L*. The diffusion eq 18 becomes
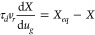
29This equation has the same form of eq 25, and the hysteresis effect shown in Figure 3f is similar to that in Figure 3c. The chemical inductor structure in eq 29 has been used to explain the inverted hysteresis^46^ and time transient responses in halide perovskite devices such as solar cells and memristors.^20,22,47,48^ We remark that the dynamic hysteresis observed in Figure 1a is clearly an inverted hysteresis. This confirms that hysteresis is due to delay of ion charging as noted in the literature.^15^

According to eq 27 if the scan rate velocity is very small, the inverted hysteresis is not onset and the system remains in quasiequilibrium as it is being charged. Quantitatively the onset of inductive hysteresis depends on the parameters *v*_*r*_, τ_*d*_, and *V*_*m*_ as described in ref (19). To evaluate the relationship, consider the following version of eq 29, valid for the tail of the distribution at *u*_*g*_ ≫ *u*_0_:
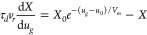
30

The solution is *X*(*u*_*g*_) = *X*_*eq*_ + Δ*X* where
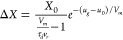
31The dynamical Δ*X* and consequently the inductive hysteresis become significant when the scan rate velocity is larger than *V*_*m*_/τ_*d*_.

However, we have shown that the series resistance of the electrolyte can produce a hysteresis effect similar to that of ion diffusion in the film. To distinguish these features, we can use other measurement techniques where the effects respond in different fashion. Here we discuss briefly the application of impedance spectroscopy for the characterization of the dynamic effects of the OECT.

To obtain the impedance response, we calculate the small signal expansion of the model eqs 8 and 18, and we apply the Laplace transform, d/d*t* → *s*, where *s* = *iω* in terms of the angular frequency ω. The result is formed by the system
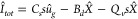
32
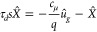
33Here the circumflex over *ŷ* denotes a small perturbation of any quantity *y*. The calculation is described in ref (42). The impedance function takes the form
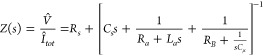
34This last function can be represented as the equivalent circuit model of Figure 4e, where the circuit elements in eq 34 have the expressions
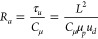
35
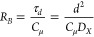
36
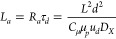
37

**Figure 4:**
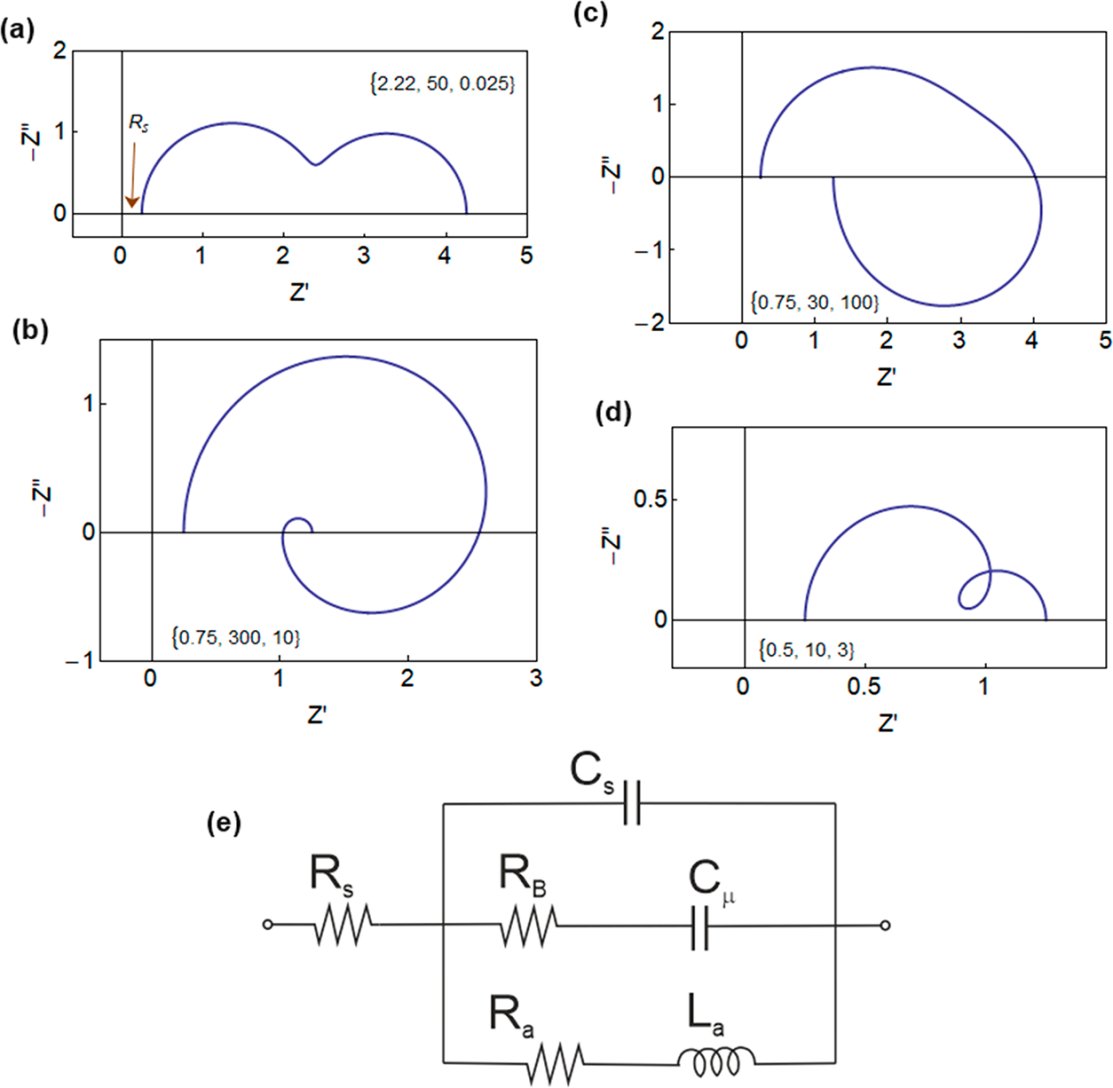
Impedance spectra for different sets of parameters. *R*_*s*_ = 0.25, *C*_*s*_ = 1, and the cases (a) *R*_*a*_ = 5, *L*_*a*_ = 0.1, *R*_*B*_ = 5, *C*_μ_ = 10; (b) *R*_*a*_ = 1, *L*_*a*_ = 10, *R*_*B*_ = 3, *C*_μ_ = 100; (c) *R*_*a*_ = 1, *L*_*a*_ = 100, *R*_*B*_ = 3, *C*_μ_ = 10; (d) *R*_*a*_ = 1, *L*_*a*_ = 3, *R*_*B*_ = 1, *C*_μ_ = 10. The indicated vectors are the time constants (*R*_*a*_^–1^ + *R*_*B*_^–1^)^−1^*C*_*s*_, *R*_*B*_*C*_μ_, *L*_*a*_/*R*_*a*_. (e) Equivalent circuit model.

We observe that *R*_*a*_ is the reciprocal of the transconductance, containing the figure of merit *C*_μ_μ_*p*_.^2^*R*_*a*_ is associated with fully electronic transport, while *R*_*B*_ is a diffusion resistance of the ions. The impedance model separates the electronic transport branch (*R*_*a*_, *L*_*a*_), which is inductive, and the ionic branch (*R*_*B*_, *C*_μ_) that charges the chemical capacitance. The inductor element *L*_*a*_ provides the coupling of ionic–electronic transport, as eq 37 contains both the kinetic transport constants for both ions (*D*_*X*_) and holes (μ_*p*_). This is because the inductor tracks the fact that if you wish to increase the lateral current by enhancing the hole density, it is actually necessary to pull the ions from the solution.

The classification of impedance spectroscopy spectra enables us to determine important parameters for hysteresis and time transient response. The effect of the series resistance *R*_*s*_ is simply a lateral displacement of the spectra as shown in Figure 4a. The double arc spectrum of Figure 4a is formed by a combination of the resistances and capacitors of the model. The rest of the spectra (panels b–d) show the negative capacitance features (i.e., the spectra move into the fourth quadrant)^22,49−51^ that are generated by the inductor element. Different cases occur according to the ordering of the characteristic times:^42,47^ (b) double curling, (c) large chemical inductor,^52^ and (d) intermediate loop. These inductive patterns have been recently reported in impedance measurement of organic transistors.^53,54^ We suggest that the inductive effects play a dominant role in the OECT and need to be systematically investigated by a combination of time domain and frequency techniques.

The impedance spectroscopy is a small perturbation measurement around a steady state, while the current measurement under cycling of the voltage produces a large voltage excursion. Despite the significant differences, the separation of the fundamental processes into equivalent circuit elements provides significant insight about hysteresis characteristics.^22^ In total, we note four relaxation phenomena that can be observed in the hysteresis equations (eqs 27 and 29) and correspondingly in the equivalent circuit of Figure 4e:

(a) (*R*_*s*_, *C*_*s*_) produces the time constant τ_*s*_.
(b) The branch (*R*_*B*_, *C*_μ_) has the time τ_*d*_ of vertical diffusion (eq 19).
(c) The inductive branch (*R*_*a*_, *L*_*a*_) has the same diffusion time τ_*d*_.^42^
(d) Additionally we observe that the cross coupling (*R*_*a*_, *C*_μ_) gives the transit time τ_*u*_ of lateral transport (eq 28).

These characteristic relaxation times can also explain different features of transient phenomena in response to voltage steps,^47,48^ but this analysis is left for a future work. The equivalent circuit of Figure 4e is inherent to the operation of the OECT, so that it forms a “minimal model” that can be complemented with additional effects as mentioned in the introduction. For example, in the case that Warburg impedances are observed, the impedance model has to be extended including spatial diffusion by solving eq 16.^55,56^

In summary, the hysteresis and more generally the time transient and impedance characteristics of the OECT have been described based on a simple model that takes into account several effects: the transversal electronic and vertical ionic currents, leading to charging of the film and ion diffusion; the electrolyte resistance and surface capacitance of the film; and the disorder effects that produce a specific form of the chemical capacitance. By combining these factors, we can classify different types of hysteresis, either capacitive or inductive, associated with the chemical inductor effect of ion diffusion in the organic film. Inductive hysteresis produces clockwise loops and capacitive hysteresis creates counterclockwise loops in the transfer current. The corresponding impedance spectra provide a valuable tool for the classification of the dominant relaxation phenomena that create the dynamical hysteresis effects.

## Supporting Information Available

Transparent Peer Review report available (PDF)
