# Supplementary material for: Hysteresis in Organic Electrochemical Transistors: Distinction of Capacitive and Inductive Effects
Source: J Phys Chem Lett. 2023 Dec 1;14(49):10951–8. doi: 10.1021/acs.jpclett.3c03062 (PMC10726359; doi:10.1021/acs.jpclett.3c03062)
Supplement: Supplementary file 1 — jz3c03062_si_001.pdf [file jz3c03062_si_001.pdf]

jz-2023-03062y.R1

Name: Peer Review Information for "Hysteresis in Organic Electrochemical Transistors: Distinction of Capacitive and Inductive Effects"

First Round of Reviewer Comments

Reviewer: 1

Comments to the Author

This is an excellent modeling work in which the author analyzes the underlying causes of hysteresis in terms of both capacitive and inductive effects in a logical manner. Since the author is a pioneer in this field, his experience in elucidating the difference between capacitive and inductive effects is quite reliable. The reviewer has only the following minor remarks on this manuscript.

1. In order to illustrate the dynamical properties of the model, the author choose equations of 18 & 19 as the equilibrium functions. The reviewer suggests the authors to add more explanation in this part.

2. The authors mentioned that $y$ denotes a small perturbation for equations 30 & 31. However, the reviewer did not see this term (y) in these two equations. Can the author check if my understanding is correct?

Author's Response to Peer Review Comments:

Journal: The Journal of Physical Chemistry Letters

Manuscript ID: jz-2023-03062y

**Reviewer 1**

Recommendation: This paper is publishable subject to minor revisions noted. Further review is not needed.

Comments:

This is an excellent modeling work in which the author analyzes the underlying causes of hysteresis in terms of both capacitive and inductive effects in a logical manner. Since the author is a pioneer in this

field, his experience in elucidating the difference between capacitive and inductive effects is quite reliable. The reviewer has only the following minor remarks on this manuscript.

Thank you very much for the positive comments

1. In order to illustrate the dynamical properties of the model, the author choose equations of 18 & 19 as the equilibrium functions. The reviewer suggests the authors to add more explanation in this part.

We have better explained the meaning of the thermodynamic function, including an important example (the Frumkin isotherm) in Eq. (9).

2. The authors mentioned that $y$ denotes a small perturbation for equations 30 & 31. However, the reviewer did not see this term (y) in these two equations. Can the author check if my understanding is correct?

   We have clarified this point: the circumflex over denotes a small perturbation of $\hat{y}$ any quantity $.y$

Format changes

1) Title: In the main manuscript file, set the title in title case, with the first letter of each principal word capitalized.

   Done

2) Abstract: Shorten the abstract to 150 words or fewer.

   Done

3) TOC Graphic: Please resize the TOC graphic per journal guidelines (2 in x 2 in).

   Done

4) References: In both the main file and the supporting information, fix the style of all references to use JPCL formatting (check all references carefully). ***JPC Letters reference formatting requires that journal references should contain: () around numbers; author names; article title (titles entirely in title case or entirely in lower case); abbreviated 2

journal title (italicized); year (bolded); volume (italicized); and pages (first-last). Book references should contain author names; book title (in the same pattern); publisher; city; and year. Websites must include date of access.

   Done

5) Graphics: One or more of your figures and tables includes a reference citation. Please confirm that this pertains only to data and not the figure itself. If it pertains to the use of a published image, permissions must be secured for any graphics NOT originally published by ACS or for Open Access content which permits reuse with credit only. Permission is needed if you are using another

publisher's or copyright owner's figures/tables verbatim, adapting/modifying them, or using them in part. If the images are from an Open Access publisher that does not require permission for reuse, please confirm.

The authors of the figure own the rights by creative common
